# Human Coronavirus NL63 Molecular Epidemiology and Evolutionary Patterns in Rural Coastal Kenya

**DOI:** 10.1093/infdis/jiy098

**Published:** 2018-03-21

**Authors:** Patience K Kiyuka, Charles N Agoti, Patrick K Munywoki, Regina Njeru, Anne Bett, James R Otieno, Grieven P Otieno, Everlyn Kamau, Taane G Clark, Lia van der Hoek, Paul Kellam, D James Nokes, Matthew Cotten

**Affiliations:** 1 Epidemiology and Demography Department, Kenya Medical Research Institute-Wellcome Trust Research Programme; 2 School of Health and Human Sciences, Pwani University, Kilifi, Kenya; 3 Faculty of Infectious and Tropical Diseases, Faculty of Epidemiology and Population Health, London School of Hygiene and Tropical Medicine, London, United Kingdom; 4 Laboratory of Experimental Virology, Academic Medical Center of the University of Amsterdam, the Netherlands; 5 Department of Medicine, Division of Infectious Diseases, Imperial College London; 6 Kymab Ltd., Babraham Research Campus, Cambridge; 7 School of Life Sciences and Zeeman Institute, University of Warwick, Coventry; 8 Wellcome Trust Sanger Institute, Hinxton, United Kingdom

**Keywords:** virus evolution, coronavirus, repeat infection

## Abstract

**Background:**

Human coronavirus NL63 (HCoV-NL63) is a globally endemic pathogen causing mild and severe respiratory tract infections with reinfections occurring repeatedly throughout a lifetime.

**Methods:**

Nasal samples were collected in coastal Kenya through community-based and hospital-based surveillance. HCoV-NL63 was detected with multiplex real-time reverse transcription PCR, and positive samples were targeted for nucleotide sequencing of the spike (S) protein. Additionally, paired samples from 25 individuals with evidence of repeat HCoV-NL63 infection were selected for whole-genome virus sequencing.

**Results:**

HCoV-NL63 was detected in 1.3% (75/5573) of child pneumonia admissions. Two HCoV-NL63 genotypes circulated in Kilifi between 2008 and 2014. Full genome sequences formed a monophyletic clade closely related to contemporary HCoV-NL63 from other global locations. An unexpected pattern of repeat infections was observed with some individuals showing higher viral titers during their second infection. Similar patterns for 2 other endemic coronaviruses, HCoV-229E and HCoV-OC43, were observed. Repeat infections by HCoV-NL63 were not accompanied by detectable genotype switching.

**Conclusions:**

In this coastal Kenya setting, HCoV-NL63 exhibited low prevalence in hospital pediatric pneumonia admissions. Clade persistence with low genetic diversity suggest limited immune selection, and absence of detectable clade switching in reinfections indicates initial exposure was insufficient to elicit a protective immune response.

## Teaser

Infections with human coronavirus NL63 are common and reinfections occur repeatedly throughout life. A subset of repeat infections show enhanced virus replication with no evidence of genotype switching, indicating that initial exposure is insufficient to elicit a protective immune response.

Acute bacterial and viral respiratory infections are a leading cause of childhood morbidity and mortality globally [1–4]. Frequently detected viruses include respiratory syncytial virus (RSV), influenza virus, parainfluenza virus, rhinovirus, human metapneumovirus, and human coronavirus [5–7]. Six coronavirus species are known to infect humans: *Severe acute respiratory syndrome coronavirus* (SARS-CoV) and *Middle East respiratory syndrome coronavirus* (MERS-CoV), associated with zoonosis and high mortality [8–11], and *Human coronavirus* (HCoV)-*NL63*, -*OC43*, -*229E*, and -*HKU1*, with higher prevalence but reduced mortality [12–15].

Human coronaviruses can infect all age groups [13, 16, 17]. Infections with HCoV-NL63, HCoV-OC43, and HCoV-229E can occur repeatedly throughout a lifetime. Descriptions of the genetic diversity of endemic HCoVs are limited and the factors that allow repeat infections by these viruses are not fully understood. Protective immune responses to HCoVs may be short lived or insufficient to block reinfection. Alternately, the virus may evolve to avoid protective immunity, with reinfection due to immune escape variants.

A better understanding of virus reinfection might reveal features for improving vaccines. The vaccine concept relies on exposure to a subacute dose of a pathogen resulting in protective immune responses [18, 19]. Although it is generally thought that host immune responses are protective against subsequent exposure to a virus, there is evidence from some pathogenic viruses that prior exposure and immune responses to a virus may actually promote greater virus infection or increased pathology in subsequent exposures to the virus [20]. For instance, antibodies were reported to enhance SARS-CoV cell entry [21, 22] and an animal model of SARS-CoV infection in African green monkeys showed increased liver pathology in immunized animals [23]. Antibody enhancement of flavivirus infection occurs in vitro [24] and there is evidence of immune responses to primary infections of dengue virus or feline coronavirus altering secondary infections [25]. For RSV, molecular studies have noted that previously circulating antigenic diversity may influence subsequent group and genotype predominance during the epidemics and this could be responsible for some of the reinfections observed in populations [26].

Respiratory virus surveillance has been carried out in Kilifi County, located in Coastal Kenya, with a continuous hospital-based arm and an intermittent community-based arm [5, 27–29]. We took advantage of 2 available cohorts with collections of upper respiratory samples to generate a set of local HCoV-NL63 partial spike and full genome sequences. During the course of a household-based community study in 2010, a pattern of coronavirus reinfection was noted. Samples from these cases were selected for detailed phylogenetic analysis.

## MATERIALS AND METHODS

### Study Population

This study used samples from (1) a prospective child inpatient (IP samples) surveillance of viral etiologies of pneumonia (2008 to 2014) at the Kilifi County Hospital (KCH) [5] and (2) a prospective household surveillance study (HH samples) conducted in a smaller geographical area within Kilifi County [29]. Study details have been previously described [5, 29–31]. The hospital pneumonia etiology study has been ongoing since 2002 and recruits children aged 0–59 months of age with signs of severe or very severe pneumonia that prompt admission. The household study recruited 483 participants from 47 households between December 2009 and June 2010, collecting nasopharyngeal flocked swabs from each household member twice weekly irrespective of symptoms. For both studies, samples were initially screened for a panel of respiratory viruses including 3 endemic coronaviruses (HCoV-229E, HCoV-NL63, and HCoV-OC43) using real-time reverse transcription polymerase chain reaction (RT-PCR) [32, 33]. A sample threshold cycle (Ct) value of <35.0 was considered positive for the target virus. The 25 pairs of samples for whole-genome sequencing were selected based on having 2 positive NL63 samples >14 days apart. For individual with multiple positive isolates in each period, the samples with the lowest Ct (highest viral load) were selected. Furthermore, to distinguish prolonged shedding from reinfection, pairs were chosen that had at least 4 NL63-negative samples in the intervening period between positive samples.

The samples in this study were collected after receiving informed written consent from each participant if ≥18 years of age or through a guardian or parent if <18 years of age and all children assented to participate. The study protocol was approved by the Scientific and Ethics Review Unit of the Kenya Medical Research Institute (KEMRI), Nairobi, and Coventry Research Ethics Committee, UK.

### Laboratory Methods

#### Viral RNA Extraction, Spike Gene Amplification, and Sequencing

Viral RNA was extracted from nasopharyngeal swab samples using QIAmp viral RNA mini kit (Qiagen) using the manufacturer’s protocol. Synthesis of cDNA from the RNA used primers targeting the S1 domain of the HCoV-NL63 spike gene (Supplementary Table 1 and Supplementary Figure 1) in a 1-step 250µL RT-PCR reaction (see Supplementary Figure 1 legend for details). The DNA products were purified using the Min Elute PCR purification kit (Qiagen) and sequenced using a ABI 3130xl (Applied Biosystems) instrument with BigDye terminator kit (Qiagen), PCR primers, and an additional 6 sequencing primers (HCoV-NL63_SF1, HCoV-NL63_SF1_RC, HCoV-NL63_SF2, HCoV-NL63_SF2_RC, HCoV-NL63_SF3, HCoV-NL63_SF3_RC; see Supplementary Table 1). Individual spike sequences were quality checked, trimmed, and assembled into larger sequence contigs using Sequencher 5.10 (Gene Codes Corporation).

### Whole-Genome Sequencing

#### Sample Preparation and Nucleic Acid Extraction

Total nucleic acid extraction was performed using previously described methods [34]. Nasopharyngeal flocked swab sample raw extracts were centrifuged for 10 minutes at 10000 × g. Nonprotected DNA in the supernatant was degraded with 20 U TURBO DNase (Ambion). Nondegraded (presumably virion-protected) nucleic acid was extracted followed by reverse transcription using nonribosomal hexamers [35]. Second-strand DNA synthesis was with 5 U of Klenow fragment (New England Biolabs) and the resulting nucleic acids were purified using phenol/chloroform extraction and ethanol precipitation.

#### Library Preparation, Sequencing, and Assembly of Short Reads

Illumina libraries were prepared for each sample. Nucleic acids were sheared to 400–500 nt, ligated to sample-specific indices, and multiplexed at 80 samples per HiSeq 2500 run, generating 2–3 million 250 nt (HiSeq) paired-end reads per sample. The raw reads were trimmed to remove residual sequencing adapters and filtered to retain reads with median Phred score >35 using QUASR v7.02 [36] and assembled into contigs using de novo assembly with SPAdes 3.10.1 [37]. Coronavirus contigs were identified with ublast [38] and a Coronaviridae protein database. Overlapping contigs were joined into full-length sequences using Geneious 8.1.8 (http://www.geneious.com/) and ambiguities were resolved by consulting the original short reads. Final quality control of genomes included a comparison of the sequences, their open reading frames and the encoded proteins with reference sequences retrieved from GenBank.

#### Comparison Datasets, Phylogenetic, and Recombination Analysis

All HCoV-NL63 sequences deposited in the GenBank encoding the S1 domain of spike gene region or the entire genome were collected from GenBank (accessed September 2017). A summary of all sequences used in this study is presented in Supplementary Table 2. Alignments were prepared using MAFFT v7.154 [39]. Phylogenetic trees were constructed in MEGA v7.0.26 [40]. The appropriate evolutionary model was determined using IQ-TREE program. Maximum likelihood methods with bootstrapping (1000 iterations) were used. The aligned sequences were analyzed for recombination using the RDP4 program.

#### K-mer Method of Genotype Classification

HCoV-NL63 genotype A and B sequence sets were prepared from GenBank plus the Kilifi HCoV-NL63 sequences. KMC3 [41] was used to identify all 30-nt sequences (k-mers) present in genotype A sequences and not in genotype B sequences and vice versa. Quality-controlled short read sequences from each sample were then classified as HCoV-NL63 genotype A or genotype B based on the read’s content of genotype A and B-specific 30-nt kmers using a threshold of 20 kmer per read as defining identity to a genotype. Results were reported as number of HCoV-NL63 reads (or fraction) classified as each genotype.

#### Accession Numbers

The HCoV-NL63 spike and full genome sequences were deposited in GenBank with accession numbers MG356413–MG356452 (spike sequences) and MG428699–MG428707 (full genome sequences).

## RESULTS

**Figure 1.**
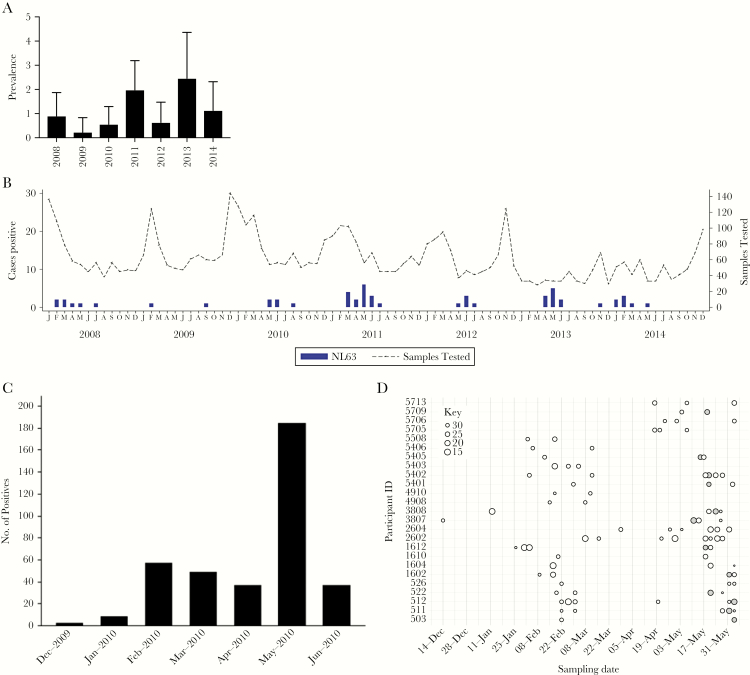
Patterns of detection of human coronavirus NL63 (HCoV-NL63) in the 2 cohorts. *A*, Prevalence of HCoV-NL63 by year in the inpatient surveillance study at Kilifi County Hospital (KCH). *B*, The frequency of detection of HCoV-NL63 by month in the inpatient surveillance study at KCH, 2008–2014. *C*, The frequency of detection of HCoV-NL63 by month in the household cohort surveillance study. *D*, Temporal patterns of HCoV-NL63 detection in the 25 community participants chosen for whole-genome sequencing. Each circle indicates the date of a positive sample; the size of the circle is inversely proportional to the real-time PCR threshold cycle (Ct) value (with scale indicating Ct to circle size is shown at the left of the panel). The grey filled circles indicate samples that yielded sequence (spike or whole genome). All positive results are shown here, while for sequencing only 2 samples were selected per individual for whole-genome sequencing (see text for details).

Of 5573 nasal samples collected in the hospital-based study between February 2008 and May 2014, diagnostic real-time RT-PCR identified 1.3% (75/5573) as HCoV-NL63 positive. Across 6 years of observation, HCoV-NL63 positive samples varied from 0.23% (2/873) for 2008 to 2.46% (11/447) for 2013 (Figure 1A) with most infections detected in February to July (Figure 1B). In the household-based community study (16918 samples), HCoV-NL63 was detected in 418 (2.5%) samples collected from December 2009 to June 2010 (Figure 1C). Among household participants, repeat infections with HCoV-NL63, HCoV-OC43, and HCoV-229E were identified in 21%, 5.7%, and 4.0% of the participants, respectively (Table 1). We selected paired samples from 25 subjects with HCoV-NL63 repeat infections for whole-genome sequencing, using the lowest Ct value sample (highest virus titer) from both first and second infections (Figure 1D). From 50 samples, 9 yielded full genomes, while 2 yielded the spike-encoding region sequences only, and all were second infection samples.

**Table 1.** Baseline Characteristics for the Household Study

	HCoV-NL631	HCoV-OC43	HCoV-229E
Number of individual infected (%)			
Single infection	163 (74.09)	215(94.30)	119 (95.97)
Second infection	46 (20.91)	13 (5.7)	5 (4.03)
Third infection	10 (4.55)	…	…
Fourth infection	1 (0.45)	…	…
Age at infection. no. (%)			
0–1 y	27 (12.27)	40 (17.54)	17 (13.71)
1–4 y	32 (14.55)	38 (16.67)	23 (18.55)
5–14 y	85 (38.64)	90 (39.47)	43 (34.68)
15–39 y	60 (27.27)	54(23.68)	31 (25.00)
40 + y	16 (7.27)	6 (2.63)	10 (8.06)
Gender of participants			
Female sex, no. (%)	121 (55.00)	130 (57.20)	70 (56.45)
Time to reinfection, days, median (IQR)			
Interval between reinfection episodes	47.00 (25, 94)	98 (87, 105)	72 (69, 101)
Frequency of households with at least 1 case of human coronavirus infection, out of the total 47 surveyed (%)	33 (70.21)	44 (93.62)	30 (63.83)
Frequency of households with at least1 case of human coronavirus reinfection (%)	12/18 (66.67)	7/18 (38.89)	3/18 (16)
Frequency of cases with coinfections with other viruses, no. (%)			
First infection	38 (79.17)	59 (96.72)	33 (89.19)
Second infection	8 (16.67)	2 (3.28)	4 (10.81)
Third infection	2 (4.17)	…	…
Presence of upper respiratory symptoms, no. (%)			
First infection	40 (72.73)	57 (95.00)	21 (91.30)
Second infection	12 (21.82)	3 (5.00)	2 (8.70)
Third infection	2 (3.64)	…	…
Fourth infection	1 (1.82)	…	…

For each virus, positive samples were determined by polymerase chain reaction (PCR) diagnostics, see Methods section.

**Figure 2.**
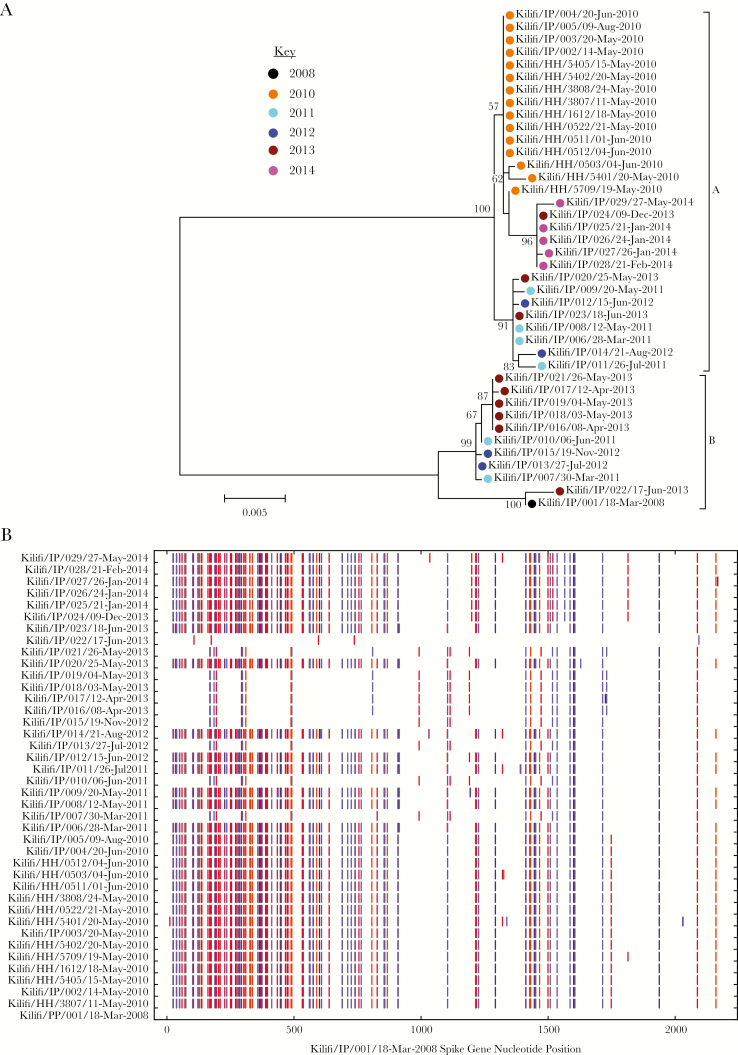

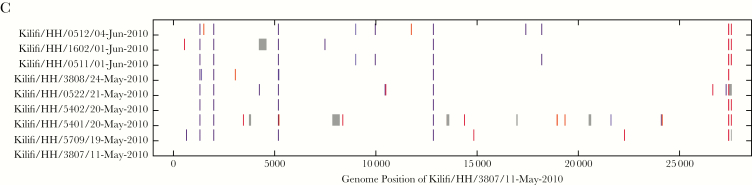
The genetic diversity of the Kilifi human coronavirus NL63 (HCoV-NL63) isolates. *A*, A maximum likelihood phylogeny of all sequenced Kilifi HCoV-NL63 strains derived from the S1 encoding region of the spike protein. The 2 main identified genotypes (A and B) are shown. The different circle colors preceding the taxon names on the phylogenetic tree depict the different years in which the samples were collected. *B*, Hiliter alignment of the Kilifi spike sequences. Changes between strains within the individual genotypes in the alignment panels are shown as colored vertical bars (orange, change to A; crimson, change to T; indigo, change to G; slateblue, change to C). *C*, A nucleotide alignment plot showing changes in the Kilifi household genomes across their length (color change coding as in *B*).

HCoV-NL63 positive samples from inpatient (IP) surveillance were subjected to spike-specific RT-PCR and dideoxy sequencing, generating 29 S1 domain sequences (2196 bp). These sequences were combined with the S1 domain from the household sequences, aligned, and a phylogeny constructed (Figure 2A and 2B). The sequences separated into 2 genotypes, A and B. For some of the observation years, both genotypes were detected in circulation (eg, 2011, 2012, and 2013) while in other years only a single genotype was detectable (Figure 2A and 2B). A nucleotide alignment of household genomes showed only a few differences distributed across their length (Figure 2C). All household study sequences belonged to genotype A. We identified the unique S1 sequences (n = 21) from the Kilifi IP-household set (n = 40) and combined them with spike sequences from other parts of the world to infer the phylogenetic placement of HCoV-NL63 circulating in Kilifi within a global context.

**Figure 3.**
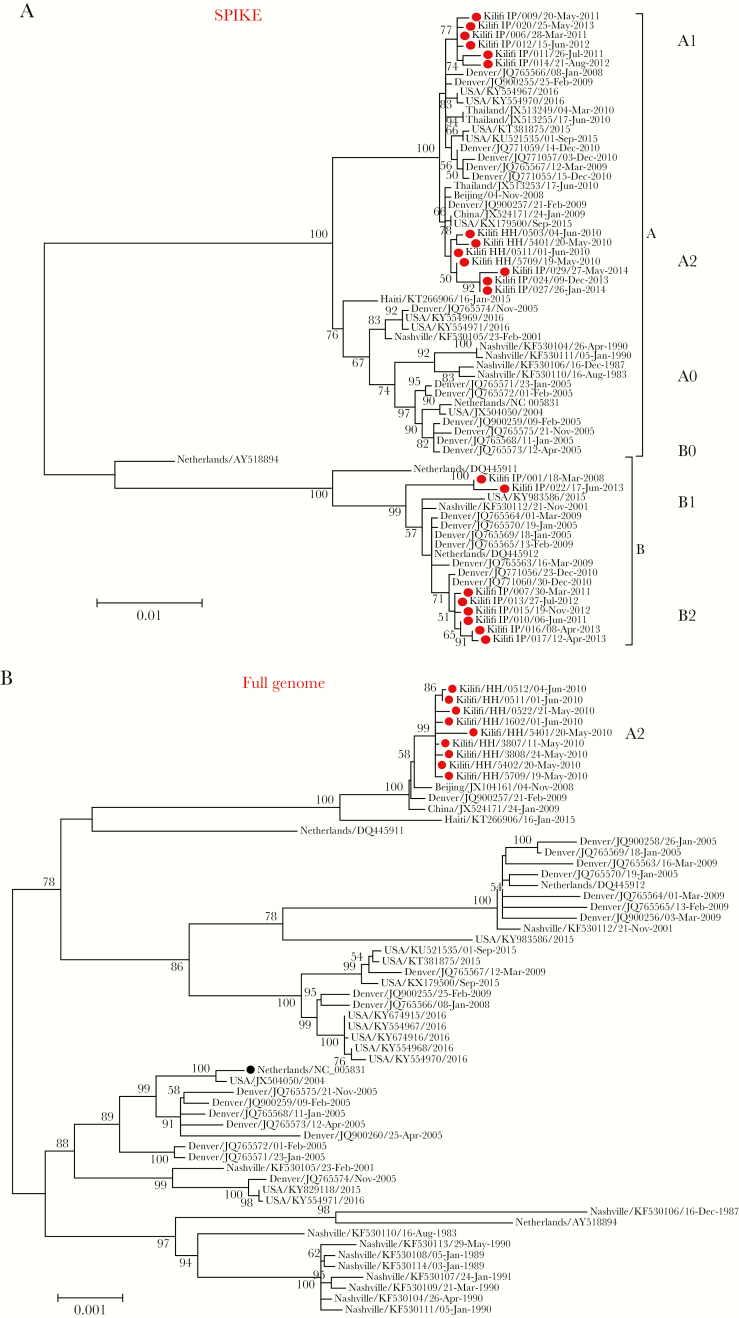

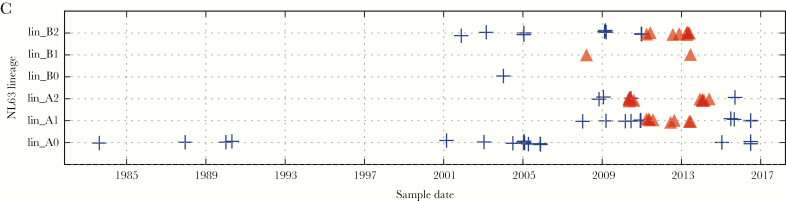
Global context of the Kilifi human coronavirus NL63 (HCoV-NL63) strains and diversity in the households genomes. *A*, A partial spike-based maximum likelihood (ML) phylogenetic tree of the combined Kilifi and global strains. *B*, A full-genome–based ML phylogenetic tree of the combined Kilifi and global strains. *C*, Local and global temporal circulation pattern of the lineages within genotype A and B. The blue symbols represent global strains while the orange symbols represent Kilifi strains. The scale bar indicates 0.01 (A) or 0.001 (B) nucleotide substitutions per site.

The global sequences (n = 63, 54 unique) originated from the United States, Haiti, Thailand, China, and the Netherlands and were isolated between 1990 and 2016. Their phylogeny including the Kilifi spike sequences confirmed the segregation of HCoV-NL63 strains in the S1 region into 2 genotypes (A and B) (Figure 3A). Similar to the Kilifi sequences, subclades within these genotypes were evident, mostly clustering by year of isolation. We assigned these subclades into lineages, A0, A1, A2, B0, B1, and B2. We constructed a phylogeny based on the Kilifi (household) and global whole-genome sequences (Figure 3B). The household genomes formed a single monophyletic group within the global phylogeny (Figure 3B). The temporal occurrence of the 6 lineages based on the spike sequences is shown in Figure 3C.

**Figure 4.**
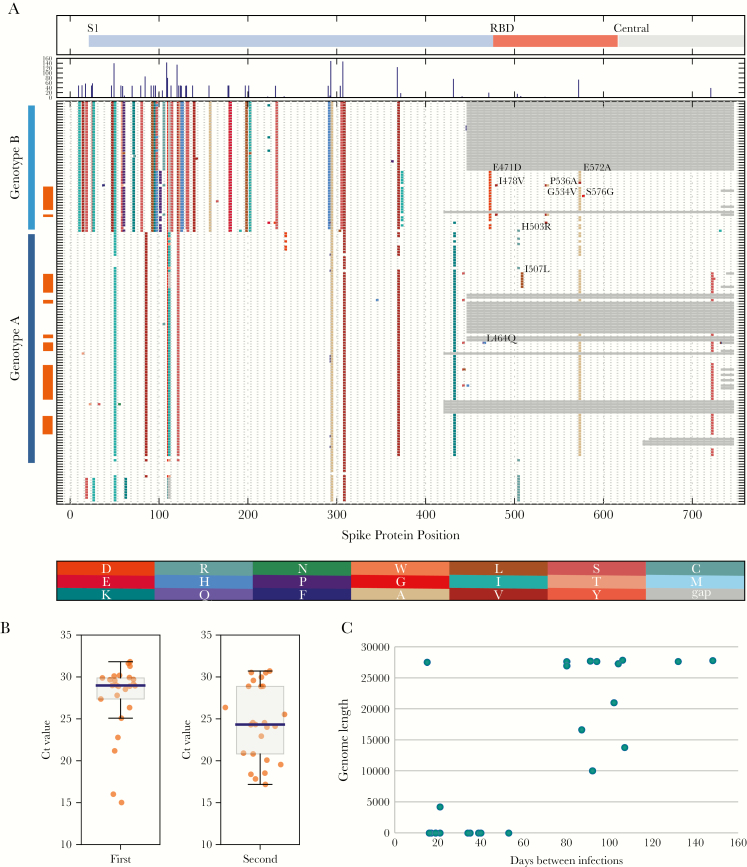
*A*, Amino acid changes in the spike S1 domain of the human coronavirus NL63 (HCoV-NL63) from all available Kilifi and global sequences. Amino acid sequences were aligned and ordered by date of sample collection (oldest at bottom, most recent at top of figure). Sequences were ordered by genotype A (dark blue bar on Y axis) or genotype B (light blue bar) and Kilifi sequences are indicated by the orange bar. Changes in the protein relative to the reference sequence (derived from the reference genome GenBank NC_005831) are indicated by colored bars; the identity of the final amino acid is indicated at the bottom of the figure. Features of the spike protein are indicated in the diagram at the top of the figure. *B*, Comparison of the threshold cycle (Ct) values of the first and second infection community samples that were selected for whole-genome sequencing, with median value (blue), interquartile range (grey box), and individual Ct values (orange circles). Samples numbers were 25 first infections, 25 second infections. The difference between the first and second infection Ct values is much greater than expected by chance (*P* value from an unpaired *t* test was .0188). *C,* The % of full genome obtained from each of the 25 second infections, plotted as a function of the length of time between the first infection sample and the second infection sample.

Global and Kilifi spike sequences were aligned and compared to the HCoV-NL63 reference strain (NC_005831) to reveal the spike amino acid differences (Figure 4A). These patterns further supported the conclusion that 2 major genotypes of HCoV-NL63 (A and B) circulated in Kilifi over the observation period (4 years for clade A, May 2010 to May 2014; 5 years for Clade B, March 2008 to June 2013).

The binding domain for the cellular receptor for HCoV-NL63 (ACE2) resides in the central portion of the spike protein, residues 476–616 [42, 43], identified by the orange horizontal band marked RBD in Figure 4A top panel. Differences in this region were marked in Figure 4A with several amino acid polymorphisms persisting in multiple samples (eg, I507L, E471D, E572A), suggesting genetic drift or possible positive advantage for these residues.

### Patterns of Coronavirus Repeat Infections

With both spike and full genome sequencing, full genome or segment sequences were successfully obtained exclusively from repeat infection samples. We examined this phenomenon in more detail.

Comparing the median Ct viral load value for first and second infections showed a large difference in the median Ct values, with second infections displaying lower Ct (higher viral loads) (Figure 4B). The difference between the 2 groups is greater than expected by chance (2-tailed *P* value = .0188) with the second exposure to the virus showing higher levels of virus replication than the previous exposure. When the yield of HCoV-NL63 genome in the second infections was plotted as a function of the time between the first and second infection, with a single exception, full genome sequence was only obtained with at least 80 days elapsing between the 2 infections (Figure 4C).

The analysis was expanded to include viral load data for 3 human coronaviruses (HCoV-NL63, HCoV-229E, and HCoV-OC43) for all positive samples in the household cohort. When plotted by sample date, 3 patterns were observed. Type 1 pattern: If the total amount of time a subject showed coronavirus-positive samples <14 days or if the subject had only a single coronavirus-positive sample the subject was considered to have a single infection. This group comprised the majority of subjects in the study (Table 2) and no conclusions about repeat infections could be made from this group. If there were at least 2 coronavirus-positive samples and the time between the first and last positive sample was ≥14 days and there were 4 intervening NL6-negative samples, the subject was considered to have a repeat (type 2) infection. A type 2A pattern was defined as having any Ct values in the second half of the period higher than any Ct value in the first half of the period. A type 2B pattern was defined as having any Ct values in the second half of the period lower than any Ct value in the first half of the period. Examples of individuals displaying the 2 patterns are shown in Supplementary Figure 2. The diagnostic results in Supplementary Figure 2*A* show individuals with low Ct values initial samples and elevated Ct values in the reinfection samples consistent with a protective effect of prior exposure to the virus. In contrast, the diagnostic results in Supplementary Figure 2*B* show the reverse pattern with at least 1 reinfection Ct value lower than any Ct value in the initial infection, indicating greater virus growth in the second infection. An analysis of the 3 coronavirus infections monitored in the cohort (HCoV-NL63, HCoV-229E, and HCoV-OC43) was performed to document the frequencies of these infection patterns across the entire cohort. HCoV-NL63 showed 21%, HCoV-229E showed 5%, and HCoV-OC43 showed 4% type 2 infections (Table 2). Among the type 2 infections, type 2A pattern (repeat infection higher Ct) was the majority pattern. However, all 3 coronaviruses showed a subset of repeat infections with higher viral loads (reduced Ct values) in the second exposure to the virus (Table 2).

**Table 2.** Observed Number of Human Coronavirus Reinfections

Virus	Total Virus-Positive Patients	Patients With Single Infections	Patients With Double Infections	Type 2A^a^	Type 2B^b^
HCoV-NL63	163	117	46 (0.28)	27 (0.59)	19 (0.41)
HCoV-OC34	215	202	13 (0.06)	9 (0.69)	1 (0.31)
HCoV-229E	119	114	5 (0.04)	4 (0.80)	1 (0.20)

^a^Defined as having any threshold cycle (Ct) value in the second half of the observation period higher than any Ct value in the first half of the period, see Results section for details.

^b^Defined as having any Ct value in the second half of the period lower than any Ct value in the first half of the period, see Results section for details.

We examined additional epidemiological data for first/second infections. All infections in the household study appeared to be mild (Table 1). Additional respiratory viruses in the Picornaviridae (6 patients), Adenoviridae (1 patient), Orthomyxoviridae (1 patient) families were detected; however, we detected no association of coinfection with severity of the second coronavirus infection.

### Comparing Sequences From First to Second Infection

One possible mechanism for repeat infection is that the second infection is with a genetically distinct virus that avoids immune responses generated by the first infection. We attempted to determine if such genotype switching occurred between first and second infections; however, the overall low viral load of the first infections made this challenging. Only a total of 9146 nucleotides of HCoV-NL63 sequence were assembled from the first infections, making it difficult to perform a comparative phylogenetic analysis across pairs. As an alternative approach we applied a more sensitive kmer method to directly genotype the HCoV-NL63 short reads from first and second infection to determine if the repeat infections involved a shift to an alternate HCoV-NL63 genotype. Training sets of all HCoV-NL63 sequences, HCoV-NL63 genotype A sequences, and HCoV-NL63 genotype B sequences (>1000 nt) was retrieved from GenBank and combined with the genotype A or B local spike sequences or the genotype A full genomes. All 30-nt kmer sequences that were present in 1 genotype and not the second were identified (see Methods and Table 3) and these genotype-specific kmers were used to classify the coronavirus reads from all 50 samples. Each read from each sample was examined for the presence of genotype-specific kmers. If 20 such kmers or more were identified with the 300-nt read, the read was classified as genotype A or B. Using this method, 259 reads were classified as HCoV-NL63 using a combined HCoV-NL63 kmer set, and 151 of the HCoV-NL63 reads could be classified by genotype and all 151 were genotype A. Second infections were all classified as genotype A, consistent with the phylogenetic analyses (see Figures 2 and 3), supporting a conclusion that genotype switching between the first and second infection is not required for reinfection.

**Table 3.** Determination of HCoV-NL63 Genotype of First and Second Infections Using kmers

	Total HCoV-NL63 reads^a^	Genotype A^b^	Genotype B^c^
First infection reads	259	151	0
First infection % genotype		100	0
Second infection reads	387489	232226	244
Second infection % genotype		99.90	0.01

^a^Total number of quality-controlled short reads mapping to any HCoV-NL63 sequence (see Methods section).

^b^Number of quality-controlled short reads identified as HCoV-NL63 genotype A by content of genotype A-specific 30 nucleotide kmers (see Methods section).

^c^Number of quality-controlled short reads identified as HCoV-NL63 genotype B by content of Genotype B-specific 30 nucleotide kmers (see Methods section).

## DISCUSSION

The HCoV-NL63 is globally ubiquitous and may have been endemic in humans for a substantial time [44]. We provide evidence of human coronavirus repeat infections in a community study and rule out a possible mechanism of genotype switching.

The prevalence of HCoV-NL63 in severe pneumonia hospital admissions of children aged less than 5 years in rural coastal Kenya was low (1.3%) and varied considerably by year, consistent with reports of HCoV-NL63 prevalence of 0.1%–6% [45–50]. HCoV-NL63 infections were detected with peak activity in May–July, coinciding with the cooler months of the year in this location (Figure 1B).

Two HCoV-NL63 genotypes were observed in Kilifi during the study period with a further diversification into lineages with a temporal clustering. Genotype A was observed in the majority of sampled infections in 2010, 2011, and 2014, while genotype B predominated in 2013. Inclusion of global sequences also supported this segregation of the HCoV-NL63 spike sequences into 2 genotypes with sublineages. Notably, the community study observed only genotype A strains (no genotype B) but it is also important to note that the study lasted only 6 months. Further studies will determine if particular genotypes contribute to more severe respiratory infections.

The Global/Kilifi spike phylogeny indicated the past circulation of up to 6 HCoV-NL63 lineages. Although the number of sequences is still too small for robust conclusions, it appears that local virus clades persist for some time: genotype A1 (2011–2013), genotype A2 (2010–2014), genotype B1 (2008–2013), and genotype B2 (2011–2013). In most cases, genomic or spike sequences from other parts of the world can be identified that were close to each Kilifi genotype. This pattern is consistent with a long period of local persistence with limited evolution of the virus, and perhaps the lack of immune pressure to change.

The observation that infection enhancement can occur after prior exposure to the virus, with strongest enhancement occurring >80 days after initial exposure (Figure 4C), is consistent with an immune response playing a role. The majority of the repeat infections showed a pattern of reduced virus replication after prior exposure, which is consistent with the vaccine principle. Also there are likely to be cases of repeat exposure to the virus where the second infection is blocked and these, of course, would not be detected in our study. However, for all 3 endemic human coronaviruses a subset of repeat infections showed enhanced virus replication in the second infection. Thus it appears that host responses to HCoV-NL63 infections vary and may be dependent on the time elapsed since previous infection (Figure 4C). In addition, we speculate that the host’s prior exposure to the virus, the host’s HLA type, the quantity of virus in inoculum, and the host’s health status may influence the outcome of the exposure. If indeed these viruses exploit the host immune response to enhance infection, this mechanism could account for the low evolutionary rate of these viruses. There would be a negative selection of amino acid changes in immune epitopes that would disrupt this enhancement.

This study had limitations. Firstly, HCoV-NL63 single infection samples failed to yield full spike region or full genome sequences, most likely related to the low virus titers. Nonetheless, we could generate sufficient signal using a kmer approach (Table 3) to conclude that genotype switching did not accompany reinfection. We do realize the limitations of the kmer approach to detect specific differences in the reinfecting virus, such as changes in immune epitopes that might accompany reinfection. Secondly, our understanding of the global migration of HCoV-NL63 was hampered by the small number of HCoV-NL63 sequences available in GenBank. The limited global HCoV-NL63 sequence data meant that we could not infer the origins of HCoV-NL63 strains circulating in Kilifi with any detail. Nonetheless, the global data combined with the new Kilifi data from this report revealed a surprising stability in HCoV-NL63 with genotypes detectable globally over 10–15 years of observation. Recent increases in virus sequence surveillance will benefit this field and will provide data for a more detailed understanding of HCoV-NL63 genetic diversity and phylogeography.

In summary, this study described HCoV-NL63 infection patterns in rural coastal Kenya. Two HCoV-NL63 genotypes circulated in Kilifi and this mirrored findings from global data. Virus lineages circulated within the community over several years, suggesting no requirement of reintroduction for persistence and hence absence of herd immunity. Reinfections with HCoV-NL63 did not require genotype switching and there were multiple cases where the second infections resulted in higher viral loads than the initial infection, revealing ineffective protective immune responses after initial exposure to the virus. Finally, the new HCoV-NL63 sequences generated here provide useful data for coronavirus surveillance, primer design, and other efforts to document the evolutionary patterns of this virus.

## Supplementary Data

Supplementary materials are available at *The Journal of Infectious Diseases* online. Consisting of data provided by the authors to benefit the reader, the posted materials are not copyedited and are the sole responsibility of the authors, so questions or comments should be addressed to the corresponding author.

Supplementary Figure 1

Supplementary Figure 2

Supplementary Figure Legends

Supplementary Tables

## Notes

***Authors’ contributions.*** P. K. K. designed and implemented the study, performed partial S1 domain gene sequencing, data analysis, and drafted the manuscript. C. A. N. helped design the study, performed data analysis, and drafted the manuscript. P. K. M. designed the household study. R. N. and A. B. conducted RT-PCR for the household study. J. R. O., G. P. O., and E. K. performed data analysis. T. K. critically reviewed the manuscript. T. G. C. helped develop the study. L. H. helped develop the full genome sequencing strategy. P. K. helped develop the sequencing strategy. D. J. N. designed the study. M. C. helped design the study, developed the sequencing strategy and assembled the full genomes, conducted data analysis, and drafted the manuscript. All authors critically reviewed the manuscript.

***Acknowledgments.*** We thank the Kilifi VEC group, field and clinical staff, for collecting the samples analyzed here and also the guardians/ parents of the children who participated in the study. We thank the Illumina sequencing team at the Sanger Institute for their support. The study is published with permission of the Director of KEMRI.

***Financial support.*** This work was supported by the Wellcome Trust, UK (grant number 102975) and the Commonwealth Distance Learning Scholarship Scheme to P. K. K. (grant number KECD-2013–54). T. G. C. is funded by the Medical Research Council, UK (grant numbers MR/K000551/1 and MR/M01360X/1, MR/N010469/1, MC_PC_15103). P. K. and M. C. were supported by Wellcome Trust core grant funding to P. K.

***Potential conflicts of interest.*** All authors: No reported conflicts of interest. All authors have submitted the ICMJE Form for Disclosure of Potential Conflicts of Interest. Conflicts that the editors consider relevant to the content of the manuscript have been disclosed.
