# Supplementary figures and images for: Human Coronavirus NL63 Molecular Epidemiology and Evolutionary Patterns in Rural Coastal Kenya
Source: J Infect Dis. 2018 Mar 21;217(11):1728–39. doi: 10.1093/infdis/jiy098 (PMC6037089; doi:10.1093/infdis/jiy098)

Suppl. Fig.1

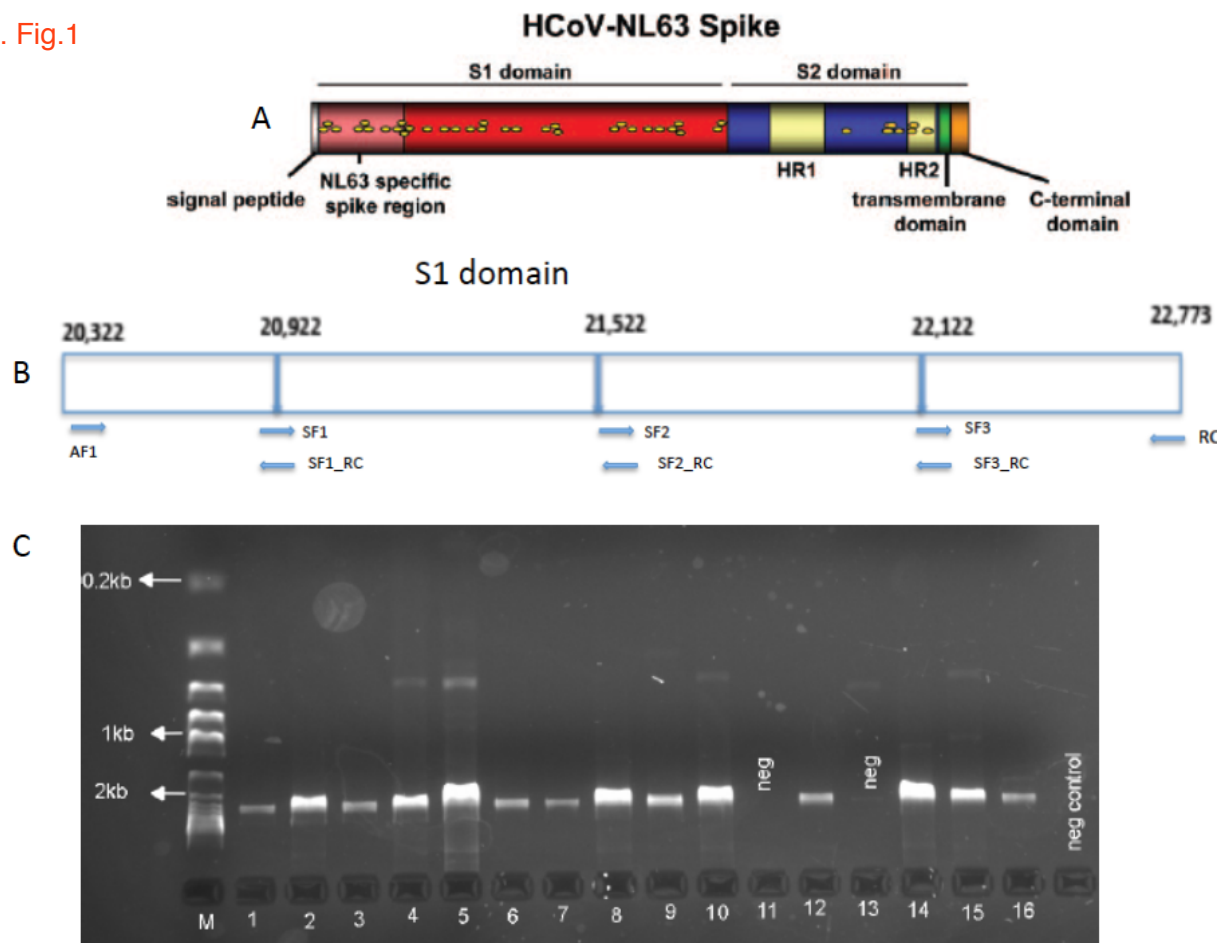

Supplement: Supplementary Figure 1 [file jiy098_suppl_supplementary_figure_1.pdf]
