# Supplementary material for: Human Coronavirus NL63 Molecular Epidemiology and Evolutionary Patterns in Rural Coastal Kenya
Source: J Infect Dis. 2018 Mar 21;217(11):1728–39. doi: 10.1093/infdis/jiy098 (PMC6037089; doi:10.1093/infdis/jiy098)

Suppl.Fig.2

A. NL63 infections (>60 days) 2nd infection Ct >1st infection Ct

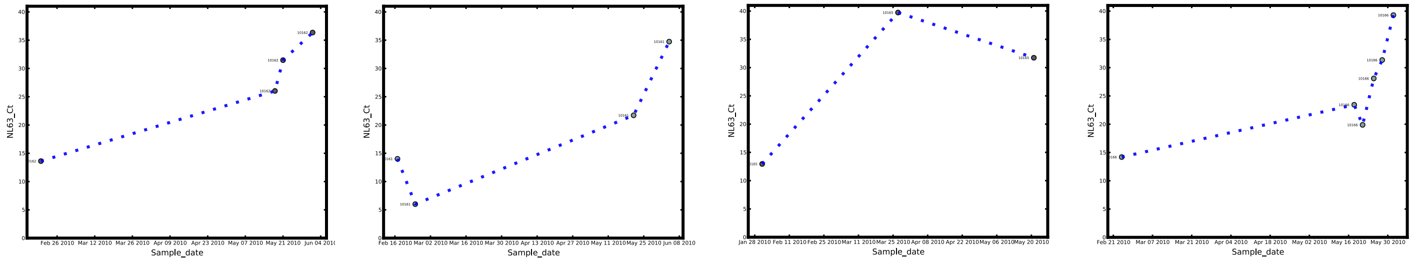

B. NL63 infections (>60 days) 2nd infection Ct <1st infection Ct

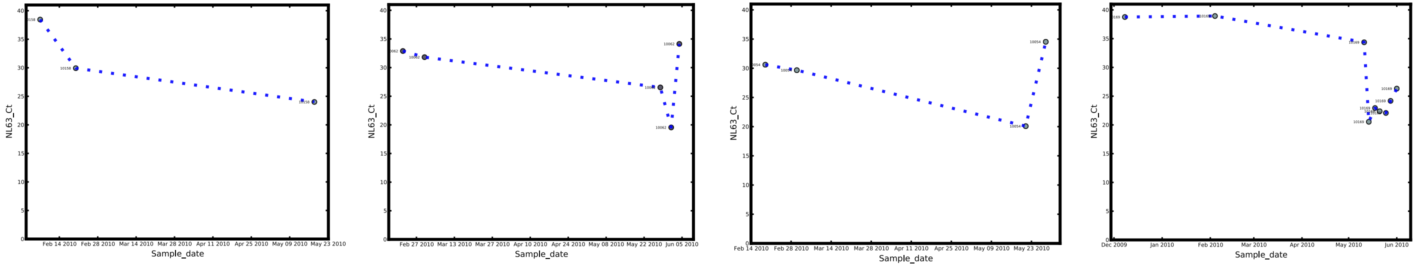

Supplement: Supplementary Figure 2 [file jiy098_suppl_supplementary_figure_2.pdf]
