# Supplementary material for: Human Coronavirus NL63 Molecular Epidemiology and Evolutionary Patterns in Rural Coastal Kenya
Source: J Infect Dis. 2018 Mar 21;217(11):1728–39. doi: 10.1093/infdis/jiy098 (PMC6037089; doi:10.1093/infdis/jiy098)
Supplement: Supplementary Figure Legends [file jiy098_suppl_supplementary_figure_legends.docx]

**Supplementary Figure 1.** Amplification strategy for the S1 domain of HCoV-NL63. Each reaction contained 5µl viral RNA, 1µl 10mM dNTP, 5µl OneStep 5x buffer (Qiagen), 1µl OneStep RT-PCR Enzyme mix (Qiagen), 0.5ul each for the forward (HCov-NL63_AF1) and reverse (HCov-NL63_RC) primers, 0.2ul of the RNaseOUT (Invitrogen) and 11.8ul nuclease free water. Reactions used a cDNA synthesis step (50°C for 30 minutes) followed by PCR cycling (95°C, 15 min, 40 cycles of (94°C, 30 seconds, 53°C, 30 seconds and 72°C 4 min) and final extension (72°C, 10 minutes). In all PCR experiments, a negative control of nuclease-free water and known positives were included. PCR products were resolved on a 2% agarose gel, stained with Red Safe (iNtRon Biotechnology, Country), and visualized under UV light. Samples with a visible band of 2.5kb were selected for sequencing. Panel A. HCoV-NL63 spike encoding region domains. Panel B. A diagram showing the binding positions of the PCR and di-deoxy sequencing primers on the S1 domian of spike protein. Panel C. An example of reverse transcriptase PCR gel result showing successful amplification of the Spike gene S1 domain. PCR amplification amplified products were resolved by agarose electrophoresis and visualized by Red Safe. Molecular size markers (M) with base pairs size are indicated to the left of the gel. Labelled 1-16 are samples, presence of a band indicates positive sample while absence means the sample was negative.

**Supplementary Figure 2.** HCoV-NL63 diagnostic values across two infections. **Panel A**: cases with Type 2A reinfection: . Defined as having any Ct values in the second half of the observation period higher than any Ct value in the first half of the period. **Panel B**: cases with Type 2B reinfection: Defined as having any Ct values in the second half of the period lower than any Ct value in the first half of the period. Ct values ordered by date of sample collection.
