# Supplementary material for: Human Coronavirus NL63 Molecular Epidemiology and Evolutionary Patterns in Rural Coastal Kenya
Source: J Infect Dis. 2018 Mar 21;217(11):1728–39. doi: 10.1093/infdis/jiy098 (PMC6037089; doi:10.1093/infdis/jiy098)
Supplement: Supplementary Tables [file jiy098_suppl_supplementary_tables.docx]

**Supplementary Table 1.** Primers used for reverse transcriptase PCR and Sanger (dideoxy) sequencing

| Primer name | Polarity | Primer sequences (5’-3’) | Tm (°C) | GC content (%) |
| --- | --- | --- | --- | --- |
| HCoV-NL63_AF1 | Forward | AARGCCACTGTTGTTGTTAC | 54.2 | 42.5 |
| HCoV-NL63_SF1 | Forward | CCTGTGTTTTTAGTGTTGTCA | 54.0 | 38.1 |
| HCoV-NL63_SF1_RC | Reverse | TGACAACACTAAAAACACAGG | 54.0 | 38.1 |
| HCoV-NL63_SF2 | Forward | TTGAAAAGTTGCAGTGTGAG | 53.2 | 40.0 |
| HCoV-NL63_SF2_RC | Reverse | CTCACACTGCAACTTTTCAA | 53.2 | 40.0 |
| HCoV-NL63_SF3 | Forward | GCCTGGTAGTTGTAATTTTCC | 55.9 | 42.9 |
| HCoV-NL63_SF3_RC | Reverse | GGAAAATTACAACTACCAGGC | 55.9 | 42.9 |
| HCoV-NL63_RC | Reverse | TAGAATTACGYRGACGAACA | 53.2 | 40.0 |

* The target for all primers was the spike gene S1 domain of HCoV-NL63

**Supplementary Table 2.** All sequences used in this study.

| **GenBank No.** | **country** | **taxon_name** | **collection**  **date** | **seq**  **length** |
| --- | --- | --- | --- | --- |
| JX524171 | China | China/JX524171/24-Jan-2009 | 24-Jan-2009 | 27538 |
| KT266906 | Haiti | Haiti/KT266906/16-Jan-2015 | 16-Jan-2015 | 27553 |
| MG356413 | *Kenya | Kilifi/IP/001/18-Mar-2008 | 18-Mar-2008 | 2192 |
| MG356414 | *Kenya | Kilifi/HH/3807/11-May-2010 | 11-May-2010 | 2195 |
| MG356415 | *Kenya | Kilifi/IP/002/14-May-2010 | 14-May-2010 | 2195 |
| MG356416 | *Kenya | Kilifi/HH/5405/15-May-2010 | 15-May-2010 | 2195 |
| MG356417 | *Kenya | Kilifi/HH/1612/18-May-2010 | 18-May-2010 | 2195 |
| MG356418 | *Kenya | Kilifi/HH/5709/19-May-2010 | 19-May-2010 | 2195 |
| MG356419 | *Kenya | Kilifi/HH/5402/20-May-2010 | 20-May-2010 | 2195 |
| MG356420 | *Kenya | Kilifi/IP/003/20-May-2010 | 20-May-2010 | 2195 |
| MG356421 | *Kenya | Kilifi/HH/5401/20-May-2010 | 20-May-2010 | 2195 |
| MG356422 | *Kenya | Kilifi/HH/0522/21-May-2010 | 21-May-2010 | 2195 |
| MG356423 | *Kenya | Kilifi/HH/3808/24-May-2010 | 24-May-2010 | 2195 |
| MG356424 | *Kenya | Kilifi/HH/0511/01-Jun-2010 | 01-Jun-2010 | 2195 |
| MG356425 | *Kenya | Kilifi/HH/0503/04-Jun-2010 | 04-Jun-2010 | 2195 |
| MG356426 | *Kenya | Kilifi/HH/0512/04-Jun-2010 | 04-Jun-2010 | 2195 |
| MG356427 | *Kenya | Kilifi/IP/004/20-Jun-2010 | 20-Jun-2010 | 2195 |
| MG356428 | *Kenya | Kilifi/IP/005/09-Aug-2010 | 09-Aug-2010 | 2195 |
| MG356429 | *Kenya | Kilifi/IP/006/28-Mar-2011 | 28-Mar-2011 | 2195 |
| MG356430 | *Kenya | Kilifi/IP/007/30-Mar-2011 | 30-Mar-2011 | 2192 |
| MG356431 | *Kenya | Kilifi/IP/008/12-May-2011 | 12-May-2011 | 2195 |
| MG356432 | *Kenya | Kilifi/IP/009/20-May-2011 | 20-May-2011 | 2195 |
| MG356433 | *Kenya | Kilifi/IP/010/06-Jun-2011 | 06-Jun-2011 | 2192 |
| MG356434 | *Kenya | Kilifi/IP/011/26-Jul-2011 | 26-Jul-2011 | 2195 |
| MG356435 | *Kenya | Kilifi/IP/012/15-Jun-2012 | 15-Jun-2012 | 2195 |
| MG356436 | *Kenya | Kilifi/IP/013/27-Jul-2012 | 27-Jul-2012 | 2192 |
| MG356437 | *Kenya | Kilifi/IP/014/21-Aug-2012 | 21-Aug-2012 | 2195 |
| MG356438 | *Kenya | Kilifi/IP/015/19-Nov-2012 | 19-Nov-2012 | 2192 |
| MG356439 | *Kenya | Kilifi/IP/016/08-Apr-2013 | 08-Apr-2013 | 2192 |
| MG356440 | *Kenya | Kilifi/IP/017/12-Apr-2013 | 12-Apr-2013 | 2192 |
| MG356441 | *Kenya | Kilifi/IP/018/03-May-2013 | 03-May-2013 | 2192 |
| MG356442 | *Kenya | Kilifi/IP/019/04-May-2013 | 04-May-2013 | 2192 |
| MG356443 | *Kenya | Kilifi/IP/020/25-May-2013 | 25-May-2013 | 2195 |
| MG356444 | *Kenya | Kilifi/IP/021/26-May-2013 | 26-May-2013 | 2192 |
| MG356445 | *Kenya | Kilifi/IP/022/17-Jun-2013 | 17-Jun-2013 | 2192 |
| MG356446 | *Kenya | Kilifi/IP/023/18-Jun-2013 | 18-Jun-2013 | 2195 |
| MG356447 | *Kenya | Kilifi/IP/024/09-Dec-2013 | 09-Dec-2013 | 2192 |
| MG356448 | *Kenya | Kilifi/IP/025/21-Jan-2014 | 21-Jan-2014 | 2192 |
| MG356449 | *Kenya | Kilifi/IP/026/24-Jan-2014 | 24-Jan-2014 | 2192 |
| MG356450 | *Kenya | Kilifi/IP/027/26-Jan-2014 | 26-Jan-2014 | 2192 |
| MG356451 | *Kenya | Kilifi/IP/028/21-Feb-2014 | 21-Feb-2014 | 2192 |
| MG356452 | *Kenya | Kilifi/IP/029/27-May-2014 | 27-May-2014 | 2192 |
| MG428699 | *Kenya | Kilifi/HH/5709/19-May-2010 | 19-May-2010 | 27438 |
| MG428700 | *Kenya | Kilifi/HH/1602/01-Jun-2010 | 01-Jun-2010 | 27439 |
| MG428701 | *Kenya | Kilifi/HH/0512/04-Jun-2010 | 04-Jun-2010 | 27439 |
| MG428702 | *Kenya | Kilifi/HH/3807/11-May-2010 | 11-May-2010 | 27434 |
| MG428703 | *Kenya | Kilifi/HH/0511/01-Jun-2010 | 01-Jun-2010 | 27440 |
| MG428704 | *Kenya | Kilifi/HH/5402/20-May-2010 | 20-May-2010 | 27438 |
| MG428705 | *Kenya | Kilifi/HH/0522/21-May-2010 | 21-May-2010 | 27350 |
| MG428706 | *Kenya | Kilifi/HH/3808/24-May-2010 | 24-May-2010 | 27437 |
| MG428707 | *Kenya | Kilifi/HH/5401/20-May-2010 | 20-May-2010 | 27437 |
| AY518894 | Netherlands | Netherlands/AY518894 | unavailable | 27555 |
| DQ445911 | Netherlands | Netherlands/DQ445911 | unavailable | 27550 |
| DQ445912 | Netherlands | Netherlands/DQ445912 | unavailable | 27535 |
| NC_005831 | Netherlands | Netherlands/NC/005831 | unavailable | 27553 |
| JQ765563 | USA | Denver/JQ765563/16-Mar-2009 | 16-Mar-2009 | 27469 |
| JQ765564 | USA | Denver/JQ765564/01-Mar-2009 | 01-Mar-2009 | 27469 |
| JQ765565 | USA | Denver/JQ765565/13-Feb-2009 | 13-Feb-2009 | 27469 |
| JQ765566 | USA | Denver/JQ765566/08-Jan-2008 | 08-Jan-2008 | 27487 |
| JQ765567 | USA | Denver/JQ765567/12-Mar-2009 | 12-Mar-2009 | 27487 |
| JQ765568 | USA | Denver/JQ765568/11-Jan-2005 | 11-Jan-2005 | 27487 |
| JQ765569 | USA | Denver/JQ765569/18-Jan-2005 | 18-Jan-2005 | 27469 |
| JQ765570 | USA | Denver/JQ765570/19-Jan-2005 | 19-Jan-2005 | 27469 |
| JQ765571 | USA | Denver/JQ765571/23-Jan-2005 | 23-Jan-2005 | 27487 |
| JQ765572 | USA | Denver/JQ765572/01-Feb-2005 | 01-Feb-2005 | 27487 |
| JQ765573 | USA | Denver/JQ765573/12-Apr-2005 | 12-Apr-2005 | 27487 |
| JQ765574 | USA | Denver/JQ765574/Nov-2005 | Nov-05 | 27487 |
| JQ765575 | USA | Denver/JQ765575/21-Nov-2005 | 21-Nov-2005 | 27487 |
| JQ771055 | USA | Denver/JQ771055/15-Dec-2010 | 15-Dec-2010 | 2195 |
| JQ771056 | USA | Denver/JQ771056/23-Dec-2010 | 23-Dec-2010 | 2192 |
| JQ771057 | USA | Denver/JQ771057/03-Dec-2010 | 03-Dec-2010 | 2195 |
| JQ771059 | USA | Denver/JQ771059/14-Dec-2010 | 14-Dec-2010 | 2195 |
| JQ771060 | USA | Denver/JQ771060/30-Dec-2010 | 30-Dec-2010 | 2192 |
| JQ900255 | USA | Denver/JQ900255/25-Feb-2009 | 25-Feb-2009 | 27485 |
| JQ900256 | USA | Denver/JQ900256/03-Mar-2009 | 03-Mar-2009 | 27455 |
| JQ900257 | USA | Denver/JQ900257/21-Feb-2009 | 21-Feb-2009 | 27456 |
| JQ900258 | USA | Denver/JQ900258/26-Jan-2005 | 26-Jan-2005 | 27383 |
| JQ900259 | USA | Denver/JQ900259/09-Feb-2005 | 09-Feb-2005 | 27502 |
| JQ900260 | USA | Denver/JQ900260/25-Apr-2005 | 25-Apr-2005 | 27459 |
| JX504050 | USA | USA/JX504050/2004 | 2004 | 27553 |
| KF530104 | USA | Nashville/KF530104/26-Apr-1990 | 26-Apr-1990 | 27410 |
| KF530105 | USA | Nashville/KF530105/23-Feb-2001 | 23-Feb-2001 | 27413 |
| KF530106 | USA | Nashville/KF530106/16-Dec-1987 | 16-Dec-1987 | 27401 |
| KF530107 | USA | Nashville/KF530107/24-Jan-1991 | 24-Jan-1991 | 27410 |
| KF530108 | USA | Nashville/KF530108/05-Jan-1989 | 05-Jan-1989 | 27410 |
| KF530109 | USA | Nashville/KF530109/21-Mar-1990 | 21-Mar-1990 | 27410 |
| KF530110 | USA | Nashville/KF530110/16-Aug-1983 | 16-Aug-1983 | 27413 |
| KF530111 | USA | Nashville/KF530111/05-Jan-1990 | 05-Jan-1990 | 27410 |
| KF530112 | USA | Nashville/KF530112/21-Nov-2001 | 21-Nov-2001 | 27395 |
| KF530113 | USA | Nashville/KF530113/29-May-1990 | 29-May-1990 | 27410 |
| KF530114 | USA | Nashville/KF530114/03-Jan-1989 | 03-Jan-1989 | 27410 |
| KT381875 | USA | USA/KT381875/2015 | 2015 | 27553 |
| KU521535 | USA | USA/KU521535/01-Sep-2015 | 01-Sep-2015 | 27553 |
| KX179500 | USA | USA/KX179500/Sep-2015 | Sep-15 | 27553 |
| KY554967 | USA | USA/KY554967/2016 | 2016 | 27523 |
| KY554968 | USA | USA/KY554968/2016 | 2016 | 27523 |
| KY554969 | USA | USA/KY554969/2016 | 2016 | 27509 |
| KY554970 | USA | USA/KY554970/2016 | 2016 | 27540 |
| KY554971 | USA | USA/KY554971/2016 | 2016 | 27550 |
| KY674915 | USA | USA/KY674915/2016 | 2016 | 27302 |
| KY674916 | USA | USA/KY674916/2016 | 2016 | 27492 |
| KY829118 | USA | USA/KY829118/2015 | 2015 | 27513 |
| KY983586 | USA | USA/KY983586/2015 | 2015 | 27489 |

*Kenya. Sequences generated in this study.
